# HISTORY OF HEALTH INSURANCE COVERAGE GAPS AND HEALTH CARE USE AFTER GAINING AGE-BASED MEDICARE COVERAGE

**DOI:** 10.1093/geroni/igae098.2455

**Published:** 2024-12-31

**Authors:** Jada Morris, Dmitry Tumin, Yan Zhang

**Affiliations:** East Carolina University, Greenville, North Carolina, United States; East Carolina University, Greenville, North Carolina, United States; East Carolina University, Greenville, North Carolina, United States

## Abstract

This study investigates the relationship between gaps in health insurance coverage prior to Medicare eligibility and subsequent changes in healthcare utilization among older adults in the United States. Medicare serves as the primary healthcare system for individuals aged 65 and above, offering access to essential medical services and security to seniors nationwide. Despite this, a significant portion of Americans aged 55–64 experience interruptions in health insurance coverage. Through an examination of Medicare enrollees with the RAND HRS 2020 dataset, we aim to determine whether experiencing coverage gaps before becoming eligible for Medicare is associated with lower utilization of preventive care services after gaining Medicare coverage. We hypothesize that individuals with a history of coverage lapses will present a decrease in attendance at primary care appointments following enrollment in Medicare. The study draws upon existing literature highlighting the challenges faced by uninsured older adults in accessing preventive care, often resorting to emergency department visits for healthcare needs. Additionally, gaps in coverage may lead to delays in diagnosis and treatment, inconsistent access to medications, and increased reliance on emergency room services post-Medicare enrollment. Our objective is to provide insights into the factors influencing healthcare utilization among older adults transitioning to Medicare, with a focus on the impact of pre-Medicare coverage lapses. Our hope is that by understanding these dynamics, policymakers and healthcare providers can develop targeted interventions to address gaps in coverage and promote equitable access to preventive care services for aging populations.

